# Does walking explain associations between access to greenspace and lower mortality?

**DOI:** 10.1016/j.socscimed.2014.02.023

**Published:** 2014-04

**Authors:** Kate Lachowycz, Andy P. Jones

**Affiliations:** University of East Anglia, School of Environmental Science, Norwich NR4 7JT, UK

**Keywords:** UK, Physical activity, Walking, Greenspace, Mortality, Geographic Information Systems (GIS)

## Abstract

Despite emerging evidence that access to greenspace is associated with longer life expectancy, little is understood about what causal mechanisms may explain this relationship. Based on social-ecological theories of health, greenspace has multifaceted potential to influence mortality but the potential alternative mediating pathways have not been empirically tested. This study evaluates relationships between access to greenspace, walking and mortality. Firstly, we test for an association between access to greenspace and self-reported levels of walking using a survey of 165,424 adults across England collected during 2007 and 2008. Negative binomial regression multilevel models were used to examine associations between greenspace access and self reported number of days walked in the last month, in total and for recreational and health purposes, after controlling for relevant confounders. Secondly we use an area level analysis of 6781 middle super output areas across England to examine if recreational walking mediates relationships between greenspace access and reduced premature mortality from circulatory disease. Results show clear evidence of better greenspace access being associated with higher reported recreational walking. There were between 13% and 18% more days of recreational walking in the greenest quintile compared with the least green after adjustment for confounders. Tests for mediation found no evidence that recreational walking explain the associations between greenspace and mortality. Futhermore, whilst the relationship between greenspace access and walking was observed for all areas, the relationship between greenspace access and reduced mortality was only apparent in the most deprived areas. These findings indicate that the association between greenspace and mortality, if causal, may be explained by mediators other than walking, such as psychosocial factors. Future research should concentrate on understanding the causal mechanisms underlying observed associations.

## Highlights

• This study examines relationships between greenspace access, walking and mortality.
• Better greenspace access was associated with higher levels of recreational walking.
• Better greenspace access was associated with lower mortality only in deprived areas.
• Walking did not meditate relationships between greenspace and mortality.

## 1 Introduction

Research into how features of the physical environment affect physical activity is part of a wider ‘social ecological’ approach to understanding health. Social ecological models seek to understand physical activity behaviours as the result of a broad spectrum of factors, including drivers at the intrapersonal, interpersonal, organisational, community and public levels (Sallis et al., 2008). These factors are represented as interacting states, as, for example, the response of individuals to features within the environment depends on their own attitudes and motivations and also those of their surrounding friends and family. Understanding these interactions helps develop effective multi-level approaches to improve health behaviours. The basic premise of social ecological models is that, along with interventions aimed at changing behaviour at an individual level, environmental change can support people to be active and make healthy choices.

Within socio-ecological literature, good access to public greenspaces, such as parks and woodland, is frequently cited as a potential health promoting resource. This conclusion is based on the principle that greenspace can be used for physical activities such as walking, cycling and sports. In addition, greenspace has wider potential influences on health. In particular, greenspace may enhance mental health (Francis et al., 2012; Ward Thompson et al., 2012) through the psychological benefits of viewing and interacting with nature (Nilsson et al., 2011) and its role of bringing people together within a social space (Maas et al., 2009a). Moreover, there are well established reciprocal links between physical activity and mental health (Penedo and Dahn, 2005) and evidence that activity in natural areas has greater psychological benefits than the equivalent exercise indoors (Coon et al., 2011) or in built environments (Mitchell, 2013). Therefore, greenspace has a multifaceted potential to influence a range of health outcomes through several theoretically plausible and interacting causal pathways.

Despite a recent proliferation of studies examining relationships between access to greenspace and health outcomes, including physical activity and markers of morbidity and mortality (for a summary see (Lachowycz and Jones, 2011; Lee and Maheswaren, 2010)) there is still little understanding about the underlying causal pathways. To address this, a theoretical framework was developed (Lachowycz and Jones, 2013) which builds upon social-ecological theories to summarise potential causal pathways between access to greenspace and health outcomes. The framework suggests how moderators, such as gender and socio-economic factors, influence the strength of relationships, as well as highlighting how mediating processes – such as use of greenspace and perceptions of the living environment – drive associations between access and both physical and psychological health outcomes.

The theoretical pathway explored in this study involves the relationship between access to greenspace, physical activity and cardiovascular mortality. The health benefits of physical activity are well established, and it is accepted that regular activity is effective in the primary and secondary prevention of several chronic diseases, including cardiovascular disease, cancer, osteoporosis and diabetes (Warburton et al., 2006). There is also compelling evidence that being physically active is associated with reduced risk of premature mortality from all causes and from cardiovascular disease (Warburton et al., 2006). Therefore, if there is a positive relationship between better greenspace access and higher levels of physical activity, it follows that people in greener areas may exhibit improved health outcomes, including lower cardiovascular mortality. Several studies have documented improved health outcomes in greener areas, including reduced cardiovascular and overall mortality (Mitchell and Popham, 2008; Takano et al., 2002; Villeneuve et al., 2012). However, no study has yet explicitly tested if physical activity may be the underlying mechanism which explains this phenomenon.

Walking is one of the predominant ways through which people gain their overall physical activity (Bauman et al., 2009) and there is good theoretical basis that environmental attributes, such as greenspace access, have an influence on levels of walking (Owen et al., 2004). Good greenspace access can facilitate walking primarily because it serves as a setting within (or through) which walking can take place and a destination which people can walk to (Bedimo-Rung et al., 2005).

Walking behaviours can be separated into those for health and recreation purposes (e.g. dog walking or leisure-time rambles) and those to get to and from places (e.g. to the shops or work). Whilst greenspace could have a supportive role for both types of walking behaviour, it is its role as an environment for recreational and health walks which seems most plausible and has received the most attention to date in studies. Nevertheless, the results from the studies examining greenspace access and walking outcomes are mixed (Lachowycz and Jones, 2011). While some international examples, many conducted in Australia (e.g. Giles-Corti and Donovan, 2002; Sugiyama et al., 2010), have documented higher levels of walking in greener areas, several studies in the UK have failed to find any significant relationship (e.g. Mytton et al., 2012; Panter et al., 2008; Foster et al., 2009).

In this study we test if there is an association between access to greenspace and self-reported levels of walking after adjustment for potential confounding factors amongst a large sample of adults across England. We include a measure of overall walking and one of walking specifically for recreational purposes. Secondly we examine the extent to which any associations between greenspace and recreational walking mediated the relationship between access to greenspace and reduced premature mortality from circulatory disease, a relationship previously documented for adults in England (Mitchell and Popham, 2008). This prior research found stronger associations between deprivation and mortality in less greenareas, so we stratify our analysis by deprivation, and also adjust for potential confounding factors such as urban-rural status.

## 2 Methods

### 2.1 Data sources

Data for this study were combined from individual (person based) and area level sources. The individual level data were sourced from the Active People Survey (APS), an annual survey organised by SportEngland and conducted by Ipsos Mori (Ipsos UK, 2007). The survey consists of a telephone questionnaire (using random digit dialling of telephone numbers) of a random sample of adults across England and collected information about participation in a range of physical activities. The survey is designed to be representative of the whole of England and large enough to be statistically robust in local areas. A minimum of 500 interviews in all Local Authorities across England was achieved, with the exception of Isles of Scilly and City of London due their small resident populations. This analysis uses the data collected between October 2007 and October 2008 (SportEngland, 2011).

In order to assign individuals to an area measure based of greenspace access and population mortality (individual mortality was not available for the APS), Ipsos Mori provided the research team with the 2001 Middle Super Output Area (MSOA) code within which each respondent resided. MSOAS are geographical units used in the UK census, of which there were 6781 in England at the 2001 Census, with a minimum population size of 5000 residents and an average of 7200 residents. The linked survey data were provided in an anonymised form without sharing the postcodes of individual participants to ensure that individuals could not be identified, thus complying with confidentiality restrictions on the data. Ethics approval was not required for this study as the analysis was based on publicly available anonymised data.

### 2.2 Measure of walking

The APS included two questions about walking: “On how many days in the last four weeks have you walked for at least 30 min?” (Respondents were asked to include all walks of that duration, but to exclude time spent walking around shops), and “How many of those days were you walking for the purpose of health or recreation, not just to get from place to place?” Two walking outcomes were generated for each survey participant, each counting the number of days reported in response to each question.

As only area based mortality was available for the mediation analysis, an area based indicator of recreational walking was also generated for each MSOA that took account of the age and sex of respondents. Indirect standardisation was used to compute the mean per capita expected number of days walked in the last 4 weeks. This was based on the age and sex profile of the respondents in each MSOA and computed using the ratio of the observed mean number of days divided by the expected mean.

### 2.3 Measure of greenspace access

Access to greenspace was measured using the Generalized Land Use Data (GLUD) 2005 dataset (CLG, 2005). This classification allocates all identifiable features from national mapping agency (UK Ordnance Survey) data into ten land use categories. One of the categories is ‘greenspace’ which includes areas such as parks, agricultural land, woodland and grassland but excludes private gardens. These data were used to compute three measures of greenspace for each MSOA. These were the percentage of land area classified as greenspace in the MSOA, the percentage classified as greenspace in MSOAs within 5 km (defined as summed total area classified as greenspace within the MSOA and other MSOAs for which the centre point fell within a 5 km radius, divided by the total area of these MSOAs), and the percentage classified as greenspace in MSOAs within 10 km, calculated using the same method.

These three alternative measures of access were used as studies have shown that the scale and method used to measure greenspace can affect the relationship with outcomes (Higgs et al., 2012).

### 2.4 Measure of mortality

The measures of premature mortality from circulatory causes (age <75 years) for MSOAs were obtained from the Association of Public Health Observatories (APHO, 2011) in the form of standardised mortality ratios (SMRs), standardised by age and sex, over the period 2006 to 2010. Mortality from circulatory causes (ICD10 I00-I99) was used because previous research had shown these causes to have the strongest associations with greenspace access (Mitchell and Popham, 2008).

### 2.5 Statistical analysis

The first part of the analysis examined associations between the three greenspace access measures and the two walking outcome measures, using individual participants in the APS as the unit of analysis. Negative binomial regression models were used as the walking outcomes were counts (days walked) and their distribution was more overdispersed than would be found in a Poisson distribution. A three level multilevel structure was used to take account of the hierarchical nature of the dataset (survey respondents nested witin MSOAs nested within Local Authorities). All analyses were carried out using MLWLIN (Rasbash et al., 2000) accessed through STATA 11 (Statcorp, 2009) using the “runmlwin” command (Leckie and Charlton, 2011).

Models were run in three stages: First the relationships between the three measures of greenspace access and the two walking variables were tested. As the relationships may not be linear, the greenspace access measures were grouped into quintiles with the first being those respondents with the worst access. Secondly, the relationships were tested with adjustment for potential individual-level covariates collected in the APS (age, gender, ethnicity, social class, car ownership, month of data collection).

Thirdly the relationships were further adjusted for MSOA-level environmental variables: Index of multiple deprivation 2010 (CLG, 2011) urban-rural classification (ONS, 2005) and population density (ONS, 2001). The index of multiple deprivation is a relative score computed from a range of indicators across seven domains of socio-economic deprivation, including income, unemployment, education and crime. The urban-rural classification categorises each MSOA as a village hamlet or isolated dwelling, a town or fringe area (part of a settlement with less than 10,000 people) or an urban area (over 10,000 population). The population density measure used was number of residents per hectare as measured in the 2011 census.

Differences in the two walking outcomes were examined across the quintiles of greenspace access, and these were expressed as Incidence Rate Ratios (IRRs) to compare the magnitude of effect size across quintiles (i.e. the ratio of mean days walked in quintiles 2 to 5 compared with the baseline quintile) and with a test for trend across the quintiles.

**Fig. 1:**
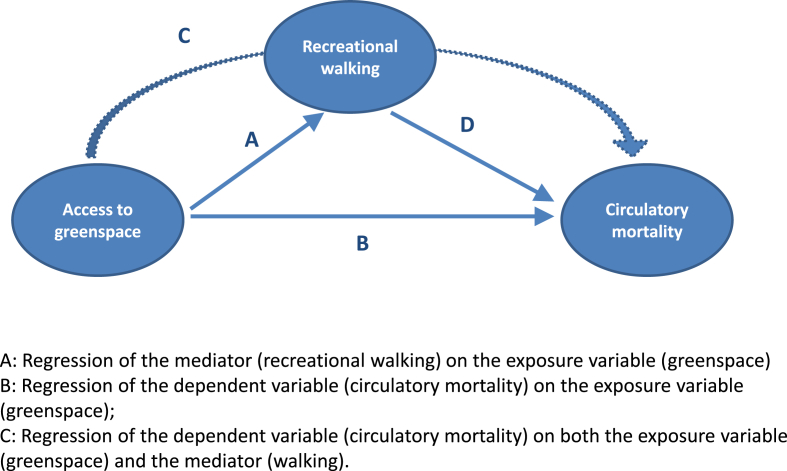
Model to test if recreational walking is a mediator in the relationship between access to greenspace and circulatory mortality.

The second part of the analysis examined if greenspace access was associated with area mortality and whether recreational walking appeared to mediate this association. It employed negative binomial regression models and was carried out in STATA, using MSOAs as the unit of analysis. The approach used to test for mediation was based on that proposed by Baron and Kenny (1986) using three regression models. The approach is illustrated in Fig. 1, with the three sequential models shown as pathways A–C on the diagram. The third model (pathway C) was also used to test if there was an association between the mediator (walking) and the dependent variable (circulatory mortality) as this would need to be present in order for mediation to occur (pathway D). There was judged to be evidence of mediation if significant associations were observed in the first and second models and the magnitude of association between greenspace and mortality was less in the third model than in the second. Perfect mediation was defined to occur if greenspace showed no association with mortality after control for walking.

In order to consider how area deprivation may modify relationships between greenspace, physical activity and mortality, the MOSA data were stratified into four deprivation quartiles based on the index of multiple deprivation 2010. The sequential Baron and Kenny test were then carried out separately for each of the four groups. All models included adjustment for urban-rural classification and population density in line with prior analysis (Mitchell and Popham, 2008). Age and sex had already been accounted for in derivation of the area mortality and walking variables.

## 3 Results

Of the 191,325 participants in the APS, 165,424 (86.5%) provided valid postcodes and so could be allocated an MSOA code and assigned measures of greenspace access.

Table 1 shows the socio-demographic factors for participants included in the analysis. Compared with the adult population of England using data from the 2011 census (ONS, 2012), survey respondents were slightly older (22.7% aged over 65 compared with 20.3% in England), more female (60.0% compared 51.3%) and less ethnically diverse (94.0% white compared 86.0%). There was an average of 24.4 respondents per middle super output area (standard deviation 15.9), with respondents from all but 8 MSOAs in England. Based on the area-level deprivation scores of the MSOAs in which respondents lived, 18.5% lived in areas classified as in the most deprived quartile of England and 32.3% lived in the most affluent quartile of areas in England.

Table 2 shows the relationship between the three greenspace access measures and the two walking outcomes. The values of the IRRs across quintiles of greenspace are shown with no adjustment, after adjustment for individual-level confounders and after additional adjustment for area-level confounders. There is clear evidence of a dose–response relationship with better greenspace access being associated with higher reporting of recreational walking, both before and after adjustment. Across the three measures of greenpace access, there were between 13% and 18% more days of recreational walking reported in the greenest quintile compared with the least green after adjustment for individual and area-level confounders.

Results for the total walking indicator were somewhat less strong (Table 2), although the highest prevalence was always recorded amongst participants living in the quintile with best access to greenspace. The strongest trend was with greenspace within 10 km of each MSOA, whereby there was a 10% higher post-adjustment reported prevalence of total walking in the greenest quintile compared with the least green.

**Fig. 2:**
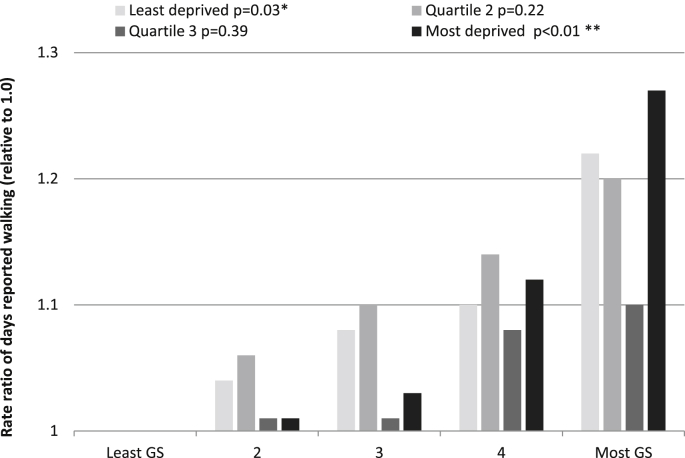
Rate ratios of days reported walking for recreation and health purposes within the last 4 weeks: By quartile of deprivation and relative to the group with the poorest access to greenspace (group 1). Test for trend: *p < 0.05, **p < 0.01.

The results from the first model of the mediation analysis, regressing the mediator (recreational walking) on the exposure variable (greenspace), are illustrated in Fig. 2. Only the findings for the 5 km measure of greenspace are presented as those from the other two measures are similar. For each of the deprivation groups, there was more reported recreational walking in greener areas. This trend was statistically significant in the most deprived group, whereby people living in greenest areas reported 27% more days with walking for recreational or health purposes compared with those in the least green areas (test for trend; *p* < 0.001).

**Fig. 3:**
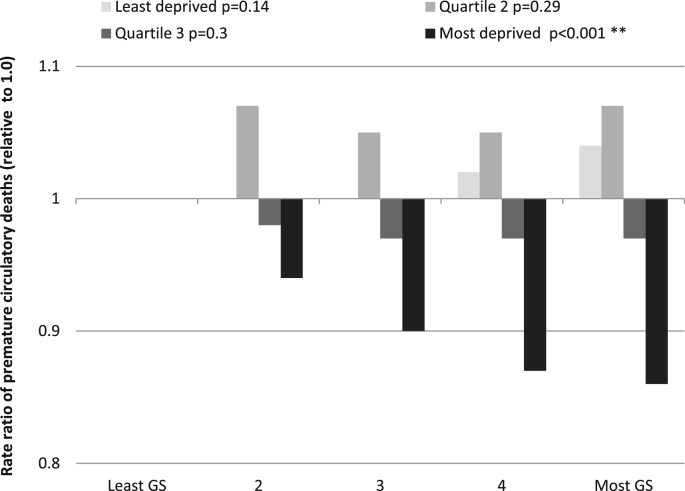
Rate ratios of premature circulatory deaths: By quartile of deprivation and relative to the group with the poorest access to greenspace (group 1). Test for trend: *p < 0.05, **p < 0.01.

The results from the second model – regressing the dependent variable (circulatory mortality) on the exposure variable (greenspace) are illustrated in Fig. 3. For the most deprived group, there was evidence of decreased premature circulatory mortality in greener areas. Relationships were strongest for the most deprived areas in which people living in the greenest areas had a 14% lower mortality rate compared with those in the least green areas (test for trend; *p* < 0.001). For the other deprivation groups, there was no clear evidence of trends in the association between greenspace and mortality.

The third model – regressing the dependent variable (circulatory mortality) on both the exposure variable (greenspace) and potential mediator (recreational walking) – did confirm a statistically significant association between recreational walking and circulatory mortality (*p* < 0.01). However, the IRRs and levels of statistical significance for the relationship between greenspace access and circulatory mortality were almost identical to those obtained in the second model. Therefore, there was no evidence that physical activity, measured by participation in walking, mediates the association between access to greenspace and mortality. For example, in the second model the IRR for the most deprived population living in areas with the most greenspace compared with the baseline least greenspace was 0.95 (0.88–1.02) for the second model and 0.96 (0.90–1.04) in the third model. Many coefficients did not change at all and there was no overall pattern of increase or decrease in values.

## 4 Discussion

This analysis of a large sample of adults across England has evaluated the relationships between greenspace access, walking and mortality from circulatory causes. One of the key gaps in knowledge highlighted by reviews of the evidence and a socio-ecological framework illustrating the relationship between access to greenspace and health was a lack of understanding around the role of mediating factors. The original contribution made by this study is that, in addition to examining the relationship between greenspace and recreational walking for a large national sample of adults, we empirically test whether recreational walking appears to explain the finding that people living in greener areas have reduced premature mortality.

Results show that people living in greener areas reported a greater number of days on which they walked for at least 30 min, even after control for potential confounding factors. These findings are consistent with some previous studies which have found associations between objectively measured greenspace access and walking, although research to date in this field has been mixed and this is the first time the relationship has been documented within the UK. The associations were stronger for recreational and health walking than walking overall, which supports the hypothesis that that this particular physical activity behaviour is likely to be encouraged by presence of greenspace in the local neighbourhood. This finding is consistent with the review of Owen et al. which highlighted that measures of walking behaviour should be specific and relevant to the feature within the environment being studied (Owen et al., 2004)

After control for confounding factors, people living in the greenest areas, based on a 5 km radius from their home MSOA, reported around 18% more days of 30 min walks undertaken for health or recreation purposes in the last month compared with those in the least green. This equates to walking around one day more per month based on the average reported 5.4 days of walking per month. Given that the UK Government recommends that people engage in five sessions of moderate–vigorous activity lasting at least 30 min per week (Department of Health (2011)), this is a relatively small contributor to achieving this target. However, there is evidence that exercise outdoors may infer additional health benefits compared with indoor settings (Coon et al., 2011), particularly for mental health, and so the health advantages of walking in green environments may be more than just their contribution to overall physical activity, especially if the walks are in natural environments.

A recent study of England adults found no association between greenspace access and overall walking or with activities hypothesised to be undertaken in greenspace (Mytton et al., 2012), but that study used a dichotomised outcome based on whether the recommended five sessions of activity had been achieved, which may explain why their results differed from ours. The other published studies within England considering objectively measured greenspace access and walking also found no association (Panter et al., 2008; Foster et al., 2009). These were both based on a sample of adults in Norwich, a relatively small city and therefore they potentially lacked heterogeneity in socio-economic factors and exposure to greenspace. A systematic review identified 50 international studies published up to 2009 which examined objectively measured greenspace access and physical activity, of which 20 found a positive association (more physical activity in greener areas), 13 found weak or mixed results, 15 found no evidence and 2 found a negative association (Lachowycz and Jones, 2011). Mixed findings may partly be a consequence of heterogeneity in approaches and methods used across studies, but may also indicate the need to better understand the causal mechanism operating in the relationship between access to greenspace and physical activity outcomes. Where results across different studies are mixed and contradictory, it is particularly important to consider what moderating factors may be operating (Sallis et al., 2008). As illustrated in our earlier theoretical framework (Lachowycz and Jones, 2013) these moderating factors can include demographic differences between the populations being studied, such as socioeconomic status, and contextual differences including psychosocial and cultural drivers. Cross cultural comparison of results may help understand this. For example, a comparison of the determinants of young people's activity between Dunedin, New Zealand, and Glasgow, Scotland, suggested that cultural differences between the two locations, rather than differences in environmental factors, were a major driver in the difference in activity levels (West et al., 2002).

The finding that recreational walking is not acting as a mediator in the relationship between greenspace access and reduced circulatory mortality indicates that this relationship is explained by other causal mechanisms. As illustrated by the theoretical framework (Lachowycz and Jones, 2013), greenspace has multifaceted potential to influence health, with the most likely potential alternative mediator being the psychosocial benefits of greenspace given that these are associated with cardiovascular health (Yusuf et al., 2004). Whilst, to our knowledge, this is the first study to test the role of recreational walking as a mediator in the relationship between greenspace access and mortality, there are indications of similar findings emerging from concurrent research. A recent study in New Zealand found that relationships between better greenspace access and lower risk of cardiovascular disease were not explained by levels of overall moderate–vigorous activity (Richardson et al., 2013), and a programme of research based in The Netherlands exploring greenspace and health concluded that stress reduction and social cohesion are more likely explanatory mechanisms underpinning relationships between greenspace access and health outcomes (Groenewegen et al., 2012). A recent exploratory study examining patterns of salivary cortisol secretion as a biomarker of stress levels found that greenspace in the living environment was associated with reduced stress, as measured by levels and patterns of cortisol secretion amongst 25 inhabitants of Dundee, Scotland. (Ward Thompson et al., 2012). The study found that this effect was not due to physical activity, pointing to the likelihood that regular visits and/or views of greenspace lie behind the association. This study demonstrated the potential to use objectively measured biological markers of mediation effects operating in practice. If used on large samples, approaches such as this could help unpick the mechanisms driving associations between greenspace exposure and health outcomes.

We confirmed the association between greenspace access and reduced cardiovascular mortality found previously (Mitchell and Popham, 2008a; Villeneuve et al., 2012) but only amongst the most deprived groups and found no evidence of physical activity, at least when measured by recreational walking, mediating this relationship. The results showed that the relationship between more greenspace and higher levels of walking held across all levels of deprivation, albeit stronger in the most deprived group than in the other groups. In contrast, the relationship between more greenspace and reduced premature mortality from circulatory causes was only present and statistically significant for the most deprived group. Given these differences in how deprivation is moderating the relationships, this is further evidence that physical activity is not acting as an underlying mechanism between access to greenspace and reduced premature mortality.

The finding that greenspace access is associated with reduced mortality only for the most deprived is consistent with some other studies which have found stronger relationships between greenspace and health outcomes for more deprived groups (e.g. Maas et al., 2009b). Potential explanations include deprived groups spending more time in their local environment (Maas et al., 2009b) or wealthier groups using local greenspace to maintain, rather than improve, their health as they incorporate other health promoting activities into their lifestyle (Lachowycz & Jones, 2012). Indeed we found that circulatory mortality amongst wealthier groups living in less green areas was similar to those in greener neighbourhoods. Factors such as larger private gardens in which to relax or greater ability to travel by car to visit leisure destinations may be important.

Consistent with our finding is that of Mitchell and Popham who reported that gradients in deprivation-related premature mortality were reduced in greener areas (Mitchell and Popham, 2008). The important consequence is that improving greenspace access could potentially reduce deprivation-related health inequalities. It is well established that more deprived populations are less physically active (Gidlow et al., 2006), have higher rates of obesity (Butland et al., 2007) and poorer health outcomes (Marmot et al., 2010). However, despite concerted public health action, deprivation-related inequalities in health outcomes persist across England (Marmot et al., 2010). The causes of these inequalities are undoubtedly multifaceted but there is increasing recognition that macro-level strategies, such as enhancing the built environment (Turrell et al., 2013) and providing greenspace, could be effective alongside individually targeted interventions (Pearce and Maddison, 2011).

Our prior theoretical model documenting relationships between greenspace and health includes a wide spectrum of mechanisms and influencing factors, many of which are poorly understood (Lachowycz and Jones, 2013). While this study has explored the role of walking as a mediator in the relationship between greenspace access and mortality, there is considerable potential for further research testing other pathways and interactions, such as how intra- and inter-personal factors moderate the relationships. One important area not explored in this study is how perceptions of access to greenspace affect relationships between objectively measured access and physical activity. Indeed, the social meaning attached to greenspace may well be a more important driver of health than merely having physical access (Macintyre et al., 2008) and it is notable that a number of studies have found poor agreement between objective and subjective measures of greenspace (e.g. Lackey and Kaczynski, 2009; Macintyre et al., 2008; Kirtland et al., 2003). Sugiyama et al. in Adelaide, Australia, found evidence that relationships between self-assessed greenspace access and self-reported physical health were mediated by higher levels of recreational walking (Sugiyama et al., 2008). While their conclusion contrasts to ours, this is representative of the mixed findings more generally in research examining the health impacts of greenspace, and demonstrates that there is still much to be explored before consensual and generalisable conclusions can be formed.

One challenge will be to determine the best scale at which to measure access to greenspace. In this study we measured the area of land classified as greenspace within the middle super output area (MSOA) in which the participants were resident and in MSOAs within a 5 km and 10 km radius. The average size of MSOAs in England is 19.2 km^2^ but the majority are considerably smaller than this: In urban areas (where 80% of MSOAs are located) the average size is 5 km^2^ and they are particularly small in inner city locations. In order to test the sensitivity of results to the scale of measurement, we generated the 5 km and 10 km measures. In particular, the 10 km measure was intended to represent an area accessible to residents for longer walks from their doorstep or easily accessed by transport. For example, people living on the fringes of an urban conurbation may choose to drive a short distance to nearby countryside in order to engage in recreational walking and so constraining their greenspace access measure just to the area most proximal to home may over-simplify the realities of human behaviour.

Despite our hypothesis that relationships may differ depending on the distance at which greenspace access was measured, results across the three measures of greenspace were actually very similar, indicating that the relationships tested were not particularly sensitive to scale in this study. This is similar to the findings of Maas et al. who found little difference in results comparing 1 km and 3 km radius distances from home (Maas et al., 2006). Others have documented greater sensitivity to scale: An analysis of RESIDE participants in Australia found that shorter distance to attractive open spaces were associated with some recreational walking, but adults with larger attractive open spaces within 1.6 km of their home were more likely to meet recommended walking levels (Sugiyama et al., 2010). We note studies are extremely heterogeneous in how greenspace access is assessed, in terms of chosen scales and also defining what constitutes “greenspace”. An emerging method, which may help researchers better understand the issue of scale, is the use of global positioning systems (GPS) to measure how far people travel from their homes to be active.

The study has a number of strengths and weaknesses. The large sample of adults was a major strength of the study, as was the use of an objectively derived measure of greenspace generated for small areas for the whole of England and linkage with mortality at a small area level. Coverage of the whole country provided good heterogeneity in greenspace exposure and sociodemographic factors. A particular strength was the attempt to examine mediation mechanisms in the relationships observed but there are caveats to using the Baron and Kenny method to test for mediation, particularly for cross sectional data (Maxwell and Cole, 2007). However, whilst there has been recent development in statistical methods to test for mediation (Emsley et al., 2010), no superior methodology is yet available which specifically fits the particular example of this dataset. There are clearly methodological limitations in using cross sectional area-level data, as testing for mediation assumes that levels of walking measured by the recent Active People Survey reflect historic levels of walking which would have contributed to levels of health and, ultimately, to premature mortality. Futhermore, the mortality data may not be based on the same people who participated in the APS. However, in the absence of longitudinal studies tracking people's exposure to greenspace and their health outcomes over the long term, the approach we used makes the best of available data.

The sampling approach excluded individuals without a landline telephone and, as with any survey, there is the risk of response bias although significant effort was made to maximise participation (MORI, 2007). A large proportion of the sample reported no recreational walking in the last four weeks (45.5%) and 7.7% of the sample reported the maximum ‘ceiling’ value of 28, meaning they walked every day. An advantage of the survey was that respondents were asked to give the number of days they had walked, rather than defining their responses into categories. Other weaknesses include that the measure of walking was self-reported, and thus subject to reporting bias. We only looked at walking, although walking is a major contributor to overall activity for most people (Bauman et al., 2009), and the survey only asked about walks of at least 30 min, thus excluding shorter bouts of activity which can have beneficial effects on health and may contribute to the overall health benefits of physical activity (Warburton et al., 2006). Participants were not asked where their walking occurred and so we cannot assume that the walking occurred within greenspace. The greenspace access data were area-based and the sizes of MSOAs vary considerably across the country. If the Active People data were available with individual based geographic identifiers (such as postcodes) and detailed greenspace mapping data were available it may be possible to derive more finely grained measures of greenspace access at individual level and potentially explore distance decay effects in the observed associations. It would also be preferable to incorporate measures of quality and type of greenspace. However, it was not feasible to generate such measures for the whole country.

The study included adjustment for socio-economic factors at an individual and area level. However, there remains the possibility of residual confounding by socioeconomic characteristics. Properties located next to greenspace or with views of nature may be the most desirable and expensive to live in or attract a certain demographic of people, but these localised and subtle differences may not be adequately captured by the measures of deprivation used. There is also the possibility of confounding by unmeasured environmental factors, such as air pollution, or by individual lifestyle variables, such as smoking, given that this is a leading cause of premature mortality. Indeed a Canadian study found that current and long term smokers live in areas with less greenspace (Villeneuve et al., 2012). There may also be selection effects, whereby people who are healthier or more active choose to live in greener areas. The finding that people living in greener areas have a lower mortality rate was consistent with previous studies but it may be that this relationship is not causal, particularly given our finding that levels of physical activity do not appear to be mediating the relationship.

In conclusion, this study represents a step forward from merely describing relationships between greenspace and health outcomes, as it considers if recreational walking is mediating this relationship. Understanding the mechanisms by which greenspace is associated with health improvement is key to inform how provision of green areas could support communities to live healthily. In England, recent changes in health service configurations has seen the public health function transfer from the National Health Service to local authorities, potentially offering greater opportunity to make evidence-based planning decisions and investments aiming at improving health and reducing health inequalities. Our study indicates that, across England, people living in greener areas engage in slightly higher levels of recreational walking. They also have slightly lower rates of premature mortality from circularly disease in the most deprived areas, although our analysis suggested recreational walking may not mediate the relationship between greenspace and mortality. Whilst our work offers support to the body of evidence that documents the health value of public greenspace, future research should concentrate on understanding the causal mechanisms underlying observed associations.

## Figures and Tables

**Table 1:** Characteristics of the survey participants.

	Number (%)	Mean (SD)
Measured at individual level		
Gender *n* = 165,424		
- Male	67,880 (40.0)	
- Female	97,544 (60.0)	
Age *n* = 165,424		55.0 (17.3)
- Working age (16–64)	127,899 (77.3)	
- Older adult (65+)	37,525 (22.7)	
Ethnic group, *n* = 159,881		
- White	150,360 (94.0)	
- Asian	4455 (2.8)	
- Black African	3156 (2.0)	
- Mixed	1202 (0.8)	
- Chinese/Other	708 (0.4)	

Social class, *n* = 156,561		
- Managerial/Professional (SEC 1,2)	69,036 (44.1)	
- Intermediate (SEC 3)	17,685 (11.3)	
- Small employers (SEC 4)	14,618 (9.3)	
- Lower supervisory/routine/never worked/unemployed (SEC5,6,7,8)	55,222 (35.3)	
Days reported walking in last 4 weeks		
- Total walking		8.3 (9.7)
- Walking for recreational and health		5.4 (8.4)
**Measured at area (MSOA) level**		
IMD deprivation		
- Most deprived (Quartile 1)	30,518 (18.5)	
- Quartile 2	40,889 (24.7)	
- Quartile 3	40,561 (24.5)	
- Least deprived (Quartile 4)	53,456 (32.3)	

Rural-urban classification		
- Urban	122,804 (75.1)	
- Town and fringe	20,276 (12.4)	
- Rural	20,344 (12.4)	

Percentage of area which is greenspace		
- Within MSOA		56.7 (26.2)
- Within 5 km		67.8 (21.4)
- Within 10 km		73.0 (19.1)

**Table 2:** Rate ratios (and 95% confidence intervals) of number of days reported walking for recreation and health purposes and in total within the last 4 weeks: By quintile of access to greenspace.

	Walking for recreation and health			Total walking		
	Unadjusted	Adjusted for individual variables^a^	Adjusted for individual and area variables^b^	Unadjusted	Adjusted for individual variables^a^	Adjusted for individual and area variables^b^
Greenspace within MSOA						
Quintile 1 (worst access)	1	1	1	1	1	1
Quintile 2	1.03 (1.00–1.06)	1.01 (0.98–1.04)	1 (0.98–1.03)	0.97 (0.95–0.99)	0.97 (0.95–0.99)	0.97 (0.95–0.99)
Quintile 3	1.12 (1.09–1.16)	1.07 (1.04-1.04)	1.05 (1.02–1.08)	0.99 (0.97–1.01)	0.99 (0.97–1.01)	0.98 (0.96–1.01)
Quintile 4	1.21 (1.18–1.25)	1.14 (1.10–1.17)	1.08 (1.04–1.11)	1.01 (0.98–1.03)	1.01 (0.99–1.03)	0.99 (0.96–1.01)
Quintile 5 (best access)	1.42 (1.37–1.46)**	1.30 (1.26–1.34)**	1.13 (1.08–1.18)**	1.08 (1.05–1.10)**	1.09 (1.06–1.11)*	1.02 (0.99–1.05)^ns^
Greenspace 5k						
Quintile 1 (worst access)	1	1	1	1	1	1
Quintile 2	1.06 (1.07–1.14)	1.05 (1.01–1.08)	1.03 (1.0–1.06)	1.00 (0.98–1.02)	0.99 (0.97–1.02)	0.99 (0.96-0.1.01)
Quintile 3	1.19 (1.14–1.23)	1.11 (1.07–1.15)	1.07 (1.04–1.11)	1.02 (0.99–1.05)	1.01 (0.98–1.02)	1.00 (0.97–1.02)
Quintile 4	1.29 (1.24–1.34)	1.18 (1.14–1.23)	1.11 (1.07–1.15)	1.05 (1.02–1.07)	1.05 (1.02–1.07)	1.02 (0.99–1.05)
Quintile 5 (best access)	1.51 (1.45–1.57)**	1.35 (1.30–1.41)**	1.18 (1.13–1.23)**	1.13 (1.10–1.17)**	1.13 (1.10–1.16)**	1.08 (1.04–1.11)**
Greenspace 10k						
Quintile 1 (worst access)	1	1	1	1	1	1
Quintile 2	1.16 (1.12–1.21)	1.10 (1.07–1.14)	1.08 (1.04–1.11)	1.03 (1.00–1.05)	1.02 (0.99–1.05)	1.02 (0.99–1.04)
Quintile 3	1.22 (1.17–1.26)	1.15 (1.11–1.19)	1.1 (1.06–1.14)	1.05 (1.02–1.08)	1.04 (1.01–1.07)	1.03 (1.00–1.06)
Quintile 4	1.27 (1.22–1.32)	1.20 (1.15–1.24)	1.12 (1.07–1.16)	1.08 (1.05–1.11)	1.07 (1.04–1.10)	1.04 (1.01–1.07)
Quintile 5 (best access)	1.46 (1.40–1.52)**	1.34 (1.29–1.40)**	1.17 (1.13–1.22)**	1.16 (1.13–1.19)**	1.15 (1.12–1.19)**	1.10 (1.06–1.14)**

Test for trend across quintiles: *p < 0.05, **p < 0.01, ns = not significant.

a Individual level variables included in model: age, gender, ethnicity, social class, car ownership, month of data collection.

b Area level variables included in model: Index of multiple deprivation 2010, urban-rural classification, population density.
